# Management of pasireotide-induced hyperglycemia in patients with acromegaly: An experts’ consensus statement

**DOI:** 10.3389/fendo.2024.1348990

**Published:** 2024-02-09

**Authors:** Sylvère Störmann, Sebastian M. Meyhöfer, Jan B. Groener, Johanna Faust, Katharina Schilbach, Jochen Seufert, Bruno Vergès

**Affiliations:** ^1^ Medizinische Klinik und Poliklinik IV, LMU Klinikum, Ludwig-Maximilians-Universität München, Munich, Germany; ^2^ Institute for Endocrinology & Diabetes, University of Lübeck, Lübeck, Germany; ^3^ German Centre for Diabetes Research (DZD), Munich-Neuherberg, Germany; ^4^ Zentrum für Diabetes und Hormonerkrankungen Neustadt, Neustadt, Germany; ^5^ Medicover Neuroendocrinology, Munich, Germany; ^6^ Klinik für Innere Medizin II, Universitätsklinikum Freiburg, Medizinische Fakultät, Albert-Ludwigs-Universität Freiburg, Freiburg, Germany; ^7^ Endocrinology Diabetics and Metabolic Disorders Department, Dijon University Hospital, Dijon, France; ^8^ French National Health and Medical Research Body Unit, Lipid-Nutrition-Cancer-1231, University of Burgundy, Dijon, France

**Keywords:** acromegaly, pasireotide, hyperglycemia, diabetes mellitus, monitoring strategy, patient management

## Abstract

Pasireotide is a somatostatin analogue for the treatment of acromegaly, a chronic condition caused by excess growth hormone. Despite the therapeutic benefits of pasireotide as a second-line treatment for inadequately controlled acromegaly, a major concern is its hyperglycemic side-effect. Here, we provide guidance on how to select appropriate patients with acromegaly for treatment with pasireotide. We summarize baseline characteristics of patients at high risk for pasireotide-associated hyperglycemia and recommend a monitoring strategy based on the risk profile. Self-monitoring of blood glucose levels (SMBG), measurements of fasting plasma glucose (FPG), postprandial plasma glucose (PPG) and regular HbA1c measurements are the foundation of our proposed monitoring approach. The pathophysiology of pasireotide-induced hyperglycemia involves decreased secretion of the incretin hormones GIP (glucose-dependent insulinotropic polypeptide) and GLP-1 (glucagon-like peptide-1). Our expert recommendations address the specific pathophysiology of pasireotide-induced hyperglycemia by recommending the incretin-based therapeutics dipeptidyl peptidase-4 inhibitors (DPP-4i) and glucagon-like peptide-1 receptor agonists (GLP-1 RA) in all appropriate patients as an alternative to first-line monotherapy with metformin. Furthermore, we emphasize the importance of adequate control of acromegaly, excellent diabetes education, nutrition and lifestyle guidance and advise to consult expert diabetologists in case of uncertainty in the management of patients with hyperglycemia under pasireotide.

## 1 Introduction

Acromegaly is a chronic systemic disease due to an excess of growth hormone (GH) and insulin-like growth factor I (IGF-I), mainly caused by a GH-secreting pituitary adenoma (somatotropinoma). Chronic GH excess leads to numerous systemic sequelae, including cardiovascular disease, osteoarthropathy, metabolic complications, respiratory disease, increased risk for some neoplasms, vertebral fractures, reduced quality of life, and hypopituitarism (1). Growth hormone affects glucose tolerance by an increase in glucose concentrations and insulin resistance due to a bimodal effect on carbohydrate metabolism: On the one hand, GH stimulates beta cell proliferation and insulin synthesis and secretion; on the other hand, it counteracts insulin action and increases lipolysis (2). GH increases glycogenolysis and thus enhances hepatic glucose production (3). Additionally, it counteracts the effects of insulin in the liver. It also impairs muscle glucose uptake and glycogen synthesis, without affecting glucose oxidation (4). Furthermore, GH promotes lipid mobilization and oxidation in adipose tissue and inhibits lipogenesis, counter-acting insulin action. The GH-induced rise in free fatty acids (FFAs) further reduces insulin sensitivity. GH also stimulates inflammation of adipose tissue and induces adipokine secretion, which is though to contribute to systemic insulin resistance in acromegaly (4). Insulin resistance (IR), hyperglycemia and diabetes are therefore common comorbidities of acromegaly and diabetes mellitus prevalence in acromegaly ranges from 16% to 56%, with some patients only having prediabetes (impaired glucose tolerance [IGT] or impaired fasting glucose [IFG]). Less than 30% of patients maintain normoglycemia (5). Despite differing pathophysiology, acromegaly-related diabetes mellitus presents similarly to type 2 diabetes mellitus (T2DM) (2).

Somatostatin, an endogenous peptide hormone, binds to all five somatostatin receptor subtypes (SSTR1 to SSTR5) and physiologically inhibits GH secretion (6). SSTR2 and SSTR5 are highly expressed in somatotroph pituitary adenomas and neuroendocrine tumors and are key targets for pharmacological treatment (7). The first-generation somatostatin receptor ligands, octreotide and lanreotide, are currently the mainstay of acromegaly medical treatment, achieving biochemical control in approximately 40% of patients and tumor shrinkage in over 60% of patients (8). Pasireotide is a second-generation somatostatin analogue (SSA) approved for the treatment of acromegaly. While the first-generation somatostatin analogues, octreotide and lanreotide, act primarily by binding to SSTR2, pasireotide binds with high affinity to the somatostatin receptor subtypes SSTR1, SSTR2, SSTR3 and SSTR5 (9, 10). Pasireotide has a 30- to 40-fold higher affinity for SSTR5 and lower affinity for SSTR2 compared with octreotide, which is why pasireotide has been attributed with potentially greater clinical efficacy in acromegaly than first-generation SSA (9, 11, 12). A study of pasireotide versus octreotide (C2305) in 358 patients with medically naïve acromegaly found biochemical disease control, defined as GH < 2.5 µg/L and IGF-I within the age-related reference interval at 12 months, significantly more often in patients treated with pasireotide (31.3% *vs* 19.2%; p = 0.007) (13). Another trial (C2402; PAOLA) showed 15-20% of patients inadequately controlled on high doses of octreotide and lanreotide achieved biochemical control after switching to pasireotide 40 mg or 60 mg (14). A retrospective single-center study found pasireotide LAR normalized IGF-I levels in ~54% of acromegaly at least partially resistant to first-generation SSA (15). Using more stringent criteria of biochemical control, in an open-label study of patients with uncontrolled acromegaly on first-generation SSA 18 of 123 patients (14.6%) achieved strict biochemical control with pasireotide (16). Current guidelines and recommendations for the treatment of acromegaly consider long-acting-release pasireotide as a second-line treatment for patients resistant to first-generation SSA and after failure of surgery (17, 18).

Despite pasireotide’s benefits as a second line treatment for uncontrolled acromegaly, one major concern stems from its hyperglycemic side effect. The mechanistic aspects of hyperglycemia induced by pasireotide have been extensively studied and may be attributed to the different binding profiles of pasireotide and octreotide to somatostatin receptors. SSTR5, highly expressed in pancreatic beta cells, inhibits insulin secretion when activated. On the other hand, activation of SSTR2 which is strongly expressed in pancreatic alpha cells mainly inhibits glucagon secretion (9, 19). Consistent with pasireotide’s lower affinity for SSTR2 and higher affinity for SSTR5, hyperglycemia is attributed to reductions in insulin secretion with only mild inhibition of glucagon secretion (11, 20–22). Pasireotide also decreases secretion of the incretin hormones GIP (glucose-dependent insulinotropic polypeptide) and GLP-1 (glucagon-like peptide-1) without altering hepatic or peripheral insulin sensitivity, rendering antihyperglycemic drugs with incretin-based mechanism of action interesting candidates justified by pathophysiology for the treatment of pasireotide-associated hyperglycemia or diabetes (20, 21). Limited clinical data and practice approaches are available on the treatment of pasireotide-induced hyperglycemia, which include frequent monitoring of glucose levels, especially during initiation of treatment, and early and aggressive antidiabetic medical treatment to prevent alterations in glucose homeostasis (23, 24). Hyperglycemia usually occurs early, usually within the first 3 months of treatment (23). However, in rare cases, hyperglycemia may occur at a later time point during pasireotide therapy (25).

**Table 1** gives an overview of the prevalence of pasireotide-induced hyperglycemia and diabetes in clinical studies in patients with acromegaly, comparing it with baseline data where possible. Due to varying study populations and methodologies, results are diverse. Generally, irrespective of baseline status, 60-90% of acromegaly patients treated with pasireotide experience hyperglycemia, and 40-70% have diabetes.

**Table 1:** Occurrence of hyperglycemia-related adverse events and diabetes in clinical studies of acromegaly patients treated with pasireotide and comparison with baseline data.

Reference	Patients receiving pasireotide	Hyperglycemia (IFG, IGT, diabetes)*		Diabetes	
		Baseline	At end of study	Baseline	At end of study^†^
*Colao et al.*, J Clin Endocrinol Metab., 2014 (13)	178	*not reported*	57.3%	*not reported*	44.4%
*Gadelha et al.*, Lancet Diabetes Endocrinol., 2014 (14)	125	83.9%	64.0%^1^	66.2%	*not reported*
*Sheppard et al.*, Pituitary, 2015 (26)	178	32.6%	62.9%	3.9%^2^	47.8%
*Tahara et al.* Endocr J., 2017 (27)	33	66.7%	66.7%	15.2%^2^	75.8%^3^
*Muhammad et al.*, Eur J Endocrinol., 2018 (28)	61	*not reported*	98.4%^2^	68.9%	77.0%
*Shimon et al.*, Endocrine, 2018 (15)	35	*not reported*	62.9%	31.4%	48.6%
*Gadelha et al.*, Front Endocrinol., 2020 (16)	123	91.1%	76.4%^2^	42.3%	62.6%
*Colao et al.*, Eur J Endocrinol., 2020 (29)	173	48.0%	69.0%	32.3%	64.3%^†4^
*Witek et al.*, Front Endocrinol., 2021 (24)	39	61.6%	92.4%	10.3%	46.2%
*Chiloiro et al.*, Endocrine., 2021 (30)	40	50.0%	85%	14.7%	35%
*Akirov et al.*, Endocrine., 2021 (31)	19	72.2%	84.2%	33.3%	64.7%
*Stelmachowska-Banaś et al.*, Pituitary., 2022 (32)	26	88.5%	96.2%	15.4%	42.3%
*Wolf et al.*, Endocr Connect., 2022 (33)	33	45.5%	90.9%	6.1%	36.4%
*Corica et al.*, J Endocrinol Invest., 2023 (34)	21	33.3%	90.5%	14.3%	66.7%
*Gadelha et al.*, J Clin Endocrinol Metab., 2023 (35)	50	64%	72%	44%	72%

Diabetes in patients with acromegaly often results in higher morbidity and mortality (36). Although hyperglycemia is a common side effect of pasireotide treatment, recommendations based on expert consensus have only been published for patients with Cushing’s disease, but not for patients with acromegaly (37, 38). Even though there are some similarities, there are inherent differences between Cushing’s disease and acromegaly and their impact on glycemic status. Thus, expert recommendations derived from consensus discussions on managing pasireotide-induced hyperglycemia in acromegaly patients are crucial and urgently needed.

## 2 Methods

In May 2022 endocrinologists and diabetologists with expertise in the treatment of patients with acromegaly convened in Munich to discuss approaches to the management of hyperglycemia in patients with acromegaly treated with pasireotide. To identify relevant data, comprehensive literature searches were conducted prior to that meeting using the PubMed database. All published articles satisfying the search terms “pasireotide” and “acromegal*” were assembled (n = 281). All titles and abstracts were screened for relevance: Articles focusing on or at least mentioning glucose metabolism in pasireotide treatment. If an article’s relevance couldn’t be determined from the title and abstract alone, the article was skimmed or read to identify potentially relevant passages. The selected articles (n = 76) were read, summarized, and critically appraised, with key findings being extracted for in-depth discussion.

The entire group received brief plenary overviews on the current state of the field and the specific topics assigned to each participant. Subsequently, the participants collectively evaluated and intensively discussed the optimal strategy and supporting evidence. In the wake of the Munich assembly, additional virtual meetings were organized using standard web conference software. At each iteration, the current state of the recommendations was summarized highlighting unresolved issues. Consensus was finally found and formulated based on the availability of evidence and its quality. Where no evidence was available, expert recommendations were agreed upon when consensus could be reached. All meeting participants approved the final recommendations.

## 3 Patient selection for the treatment of acromegaly with pasireotide

Specific indicators may predict the response to first-generation somatostatin analogues or pasireotide, summarized in **Table 2** (12, 18). The efficacy of somatostatin analogs in somatotroph adenomas is influenced by SSTR2 and SSTR5 expression, consistent with differences in the affinity of pasireotide compared with first-generation SSA. While high SSTR2 density is a good predictor of first-generation SSA efficacy, a high SSTR5/SSTR2 ratio favors pasireotide (39, 40). A hypointense T2-weighted MRI (magnetic resonance imaging) signal of somatotroph adenomas indicates a high percentage of IGF-I reduction with first-generation SSA treatment, whereas hyperintense T2 signals correlate with positive pasireotide response (40, 41). Granulation patterns of somatotroph adenomas are related to T2-weighted MRI signals and may also be used to predict the response to SSA: densely granulated somatotropinomas typically show hypointense T2 signals, whereas sparsely granulated ones tend to show hyperintense signals, indicating potential pasireotide effectiveness (18). Low AIP (aryl hydrocarbon receptor-interacting protein) expression or highly mutated AIP tumors may respond better to pasireotide than first-generation SSA (42). Furthermore, low expression of E-cadherin and high Ki-67 expression favor pasireotide (12, 18, 43, 44). Additionally, GH-secreting adenomas with mutations in the stimulatory G-protein alpha subunit, showing higher SSTR5 expression, may be more responsive to pasireotide (45). Currently, there is a lack of comprehensive studies focusing on the factors mentioned, making them merely potential considerations. Although imaging is widely accessible, the analysis of tumor samples seldomly includes the above-mentioned factors such as SSTR staining and E-cadherin expression. Consequently, clinicians face challenges in selecting patients based on these markers and need to rely on imaging and patient history.

**Table 2:** Characteristics of somatotroph adenomas predictive of a good response to pasireotide.

Characteristics of somatotroph adenoma	Indicating responsiveness to pasireotide
SSTR5/SSTR2 expression ratio	high
T2-weighted MRI signal	hyperintense
Granulation pattern	sparsely granulated
AIP expression	low
AIP mutation status	high
Expression of E-cadherin	low
Expression of Ki-67	high

Clinical benefits of pasireotide could be seen in patients with headaches unresponsive to first-generation SSA and young patients with tumor growth despite treatment (12, 46).

Given the range of characteristics of somatotrophic adenoma that influence the response to the various SSA, it seems reasonable to assume that tumors that respond poorly to first-generation SSA are more likely to have characteristics that indicate responsiveness to pasireotide. Patients with low response to first-generation SSA should therefore be considered good candidates for treatment with pasireotide.

## 4 Risk stratification

When considering treatment of acromegaly patients with pasireotide, we suggest stratifying patients by baseline risk status for worsening of glucose control. Risk factors for pasireotide-associated hyperglycemia were identified in an analysis examining baseline characteristics and the occurrence and management of hyperglycemia during treatment with pasireotide for acromegaly in two prospective clinical trials, C2305 and C2402 (PAOLA) (47). **Table 3** summarizes baseline characteristics of patients with acromegaly who are at high or low risk for pasireotide-associated hyperglycemia (47): Patients are considered at higher risk if above the age of 40, obese, [pre-]diabetic, and/or having a history of dyslipidemia/hypertension. Accordingly, normal-weight and metabolically healthy young patients are considered being at lower risk. Therefore, it is crucial to recognize preexisting abnormal glucose metabolism and, if necessary, initiate and/or adjust antidiabetic treatments before initiating therapy with pasireotide (37). Clinicians who are not familiar with diabetes therapy should seek expert help.

**Table 3:** Baseline characteristics that determine the risk for pasireotide-associated hyperglycemia.

Baseline characteristics of patients at high or low risk for pasireotide-associated hyperglycemia (47)	
High risk	Low risk
Age ≥ 40 years	Young patients (< 40 years)
BMI ≥ 30 kg/m²	Normal weight
Prediabetes or diabetes, preferably assessed by oGTT	Metabolically healthy
History of dyslipidemia	
History of hypertension	

Despite the identification of some predictive factors for the development of pasireotide-induced hyperglycemia, it should be noted that hyperglycemia under pasireotide treatment can also be observed in patients without known risk factors (14).

Current diabetes classifications have shortcomings and newer cluster-based classifications have been proposed (48). Arguments in favor of these newer classifications are manifold. Firstly, they allow for personalized treatment plans, recognizing the unique hyperglycemia patterns in diverse individuals. Secondly, these classifications enhance understanding of the specific pathophysiological variations of hyperglycemia, which might be especially helpful in a context such as acromegaly where the underlying pathophysiology per se is different from “standard diabetes”. Thirdly, they can help predict individual responses to treatment, guiding preemptive or intensified glucose-lowering strategies. However, these classifications are not widely established and validated yet in clinical practice and therefore should not be used for clinical care at this point. Furthermore, current routine screening of acromegaly patients does not usually collect the data necessary for such classification. In the context of emerging awareness of pathophysiological peculiarities of hyperglycemia in acromegaly more research in this domain is needed and possibly the development of a specific classification warranted.

## 5 How to monitor for hyperglycemia

As worsening of glycemic control is frequently observed in patients with acromegaly treated with pasireotide, glycemic control should be monitored at the beginning and during follow-up of pasireotide therapy. Self-monitoring of blood glucose (SMBG) is an important cornerstone in monitoring glycemic control. A potential obstacle to frequent self-monitoring of blood glucose levels is that self-testing in patients who are at low risk of hyperglycemia may not be reimbursed by health insurers. Compliance may also be poor, particularly in patients without hyperglycemia, if self-testing must be performed too frequently.

In patients treated with pasireotide, fasting plasma glucose (FPG) and HbA1c levels tend to increase during the first 1-3 months of treatment and stabilize thereafter (23). Especially post-prandial glucose (PPG) measurements are important, as pasireotide-associated hyperglycemia is related to decreases in glucose-stimulated insulin secretion and incretin hormone response which should be most evident after a meal (21). Therefore, SMBG is particularly important in the first three months after starting therapy. SMBG can subsequently be replaced by regular HbA1c measurements. In low-risk patients with no worsening of glycemic control, self-measurement of blood glucose once every two weeks is considered sufficient (FPG and PPG). In high-risk patients who do not have elevated blood glucose levels, weekly blood glucose self-monitoring (FPG and PPG) is recommended in the first three months. In patients with pre-existing hyperglycemia we recommend daily SMBG measurements with at least one FPG and one PPG, ideally though as multiple-point profiles. When possible and economically feasible, high-risk patients should temporarily be equipped with devices allowing continuous glucose monitoring to detect elevated blood glucose levels early and determine deviations from the time in range precisely. This approach offers the advantage to detect glycemic alterations earlier than the use of point measurements with greater ease of use for the patients. Flash glucose monitoring devices may be an economically less challenging alternative. If worsening of blood glucose control is observed, further action should ideally be decided in consultation with a diabetologist. In the situation of development of impaired glucose tolerance (IGT) and/or impaired fasting glucose (IGT) with FPG >100 mg/dl (5.6 mmol/l) and/or 2h PPG >140 mg/dl (7.8 mmol/l) and/or an HbA1c rising to or above 5.7% (38.8 mmol/mol), antidiabetic treatment based on lifestyle recommendations such as nutrition counselling and exercise motivation should be initiated. At the latest, antidiabetic treatment should be initiated when diagnostic criteria of manifest diabetes mellitus are fulfilled with FPG values greater than 126 mg/dl (7.0 mmol/l), blood glucose levels greater than 200 mg/dl (11.1 mmol/l) measured at random or an HbA1c ≥ 6.5% (48 mmol/mol). In general, all patients should be informed of the risk of occurrence of hyperglycemic deviations with pasireotide treatment, ideally explaining basic pathophysiological principles.

During treatment with pasireotide HbA1c measurements should be routinely performed every three months and at least with each IGF-I measurement. Regular HbA1c monitoring is important to detect late-onset hyperglycemic alterations, as there is anecdotic evidence of development of diabetes even after several years of pasireotide treatment (25).

**Figure 1:**
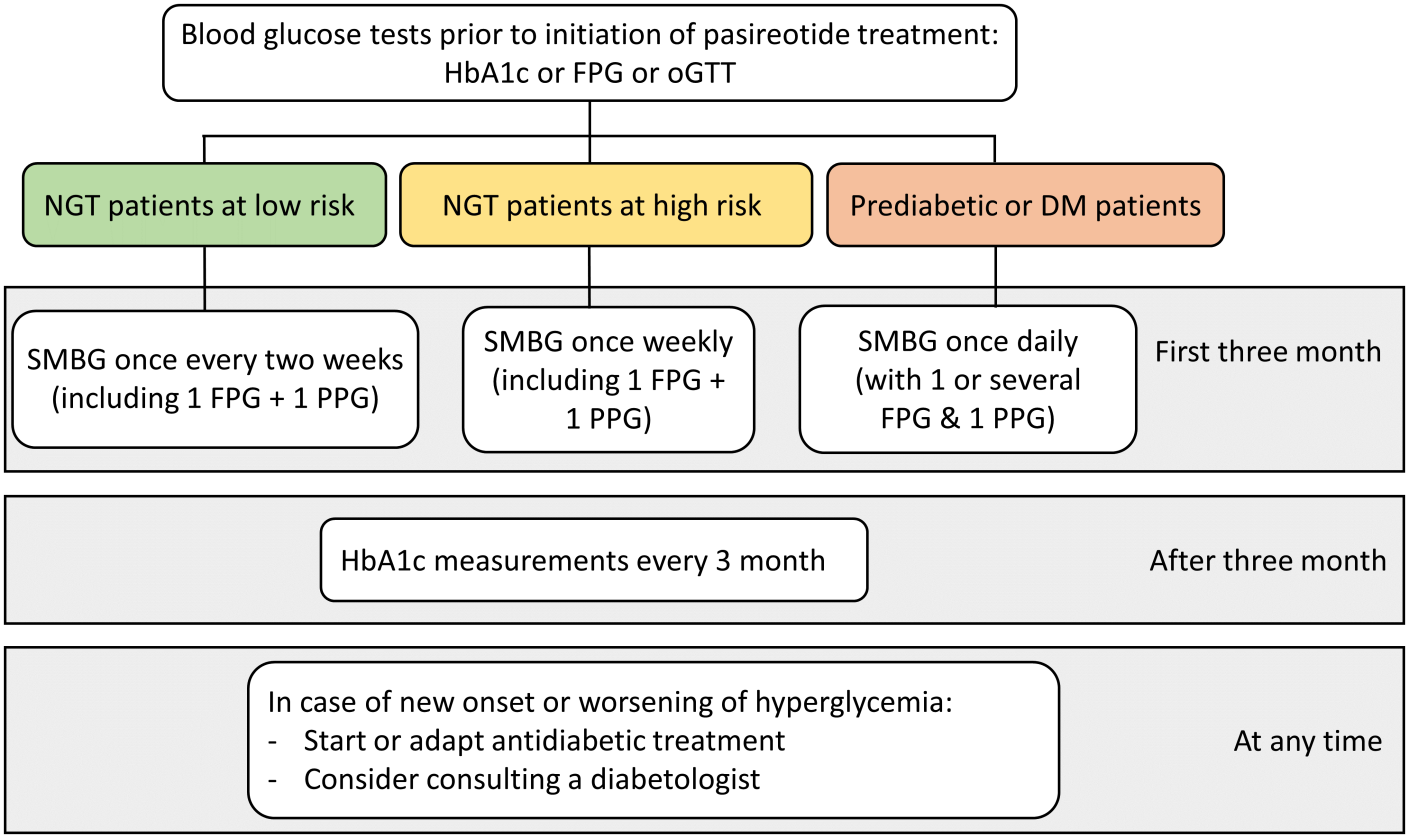
Recommendations on the monitoring of hyperglycemia in patients with acromegaly treated with pasireotide. (DM, Diabetes mellitus; FPG, Fasting plasma glucose; HbA1c, Glycated hemoglobin; NGT, Normal glucose tolerance; oGTT, Oral glucose tolerance test; PPG, Postprandial plasma glucose; SMBG, self-monitoring of blood glucose).

A summary of the recommendations on the monitoring of hyperglycemia in patients with acromegaly treated with pasireotide are displayed in **Figure 1**.

## 6 Treatment of hyperglycemia in acromegaly patients before and during pasireotide therapy

Adequate treatment of acromegaly is the priority in all patients. As long as an excess of GH is not adequately treated, the baseline for glucose metabolism will likely be suboptimal. All patients with impaired metabolic control during pasireotide therapy should receive diabetes education and nutrition and lifestyle guidance, regardless of whether they are still achieving their individual targets. Education and guidance should include physical activity, healthy sleep, high-quality nutrition, and eating patterns that result in weight loss where appropriate (49). Glycemic targets should be based on an individualized approach for each patient, considering individual risk, life circumstances, and comorbidities. This can be guided by T2DM consensus statements and guidelines (49, 50).

It is recommended that, if there is uncertainty in the management of hyperglycemia associated with pasireotide therapy, an expert diabetologist should be consulted for interdisciplinary treatment approaches. If possible, a panel of endocrinologists, diabetologists, and dieticians/diabetes educators should be scheduled to discuss cases of secondary diabetes.

Metformin should be introduced as a first-line medication when pharmacotherapy for hyperglycemia is necessary. Although metformin does not directly address the pathophysiology of pasireotide-induced hyperglycemia, there is sufficient evidence for a response to metformin. Many patients with pasireotide-induced diabetes can be controlled with metformin monotherapy (13, 14, 51). In addition, acromegaly patients are often overweight, which makes them good candidates for metformin since this medication does not promote further weight gain (47, 49).

High cardiovascular (CV) or renal risk impacts on the choice of antidiabetic therapy, thus evaluation of risks should be performed at the start of therapy (49). Since patients with acromegaly often have a high CV risk, they are prime candidates for early dual medical therapy with proven effects on cardiovascular and renal outcomes. For such patients, the positive effects on cardiovascular risk of some glucagon-like peptide-1 receptor agonists (GLP-1 RA) is particularly valuable. It’s important to recognize that not all GLP-1 RAs are identical in their efficacy. For instance, semaglutide generally shows greater glycemic control and weight loss benefits compared to dulaglutide or liraglutide. Moreover, certain GLP-1 RAs, like exenatide and lixisenatide, have not demonstrated significant cardiovascular benefits. GLP-1 RA directly target the reduced incretin secretion, which is a known mechanism of pasireotide-induced hyperglycemia (20, 21). Therefore, our expert consensus is that GLP-1 RA should be the preferred option for all suitable patients and could also be considered as a first-line treatment. We suggest prioritizing GLP-1 RA with proven superior efficacy. However, it is crucial to note the potential gastrointestinal side effects associated with GLP-1 RA, which are non-trivial in clinical practice. GLP-1 RA may also be helpful to reduce body weight if needed (49).

In patients with low CV risk, the use of dipeptidyl peptidase 4 inhibitors (DPP4i) can be considered as they neither significantly reduce nor increase cardiovascular events in most patients. DPP4i are easy to use and have few side effects, but they only indirectly affect the decreased incretin effect caused by pasireotide, as they prolong the half-life of endogenous GLP-1 but do not compensate for the decreased secretion (49). However, clinical data show promising effects of DPP4i therapy in this situation. This highlights the relevance of considering DPP4 inhibitors as a viable alternative to GLP-1 RA when gastrointestinal side-effects in the latter pose a considerable burden on patients. A recent multicenter, randomized, open-label, phase IV trial evaluated incretin-based therapy (DPP-4i followed by a GLP-1 RA) versus insulin for the treatment of pasireotide-associated hyperglycemia in adults with acromegaly (n = 190) or Cushing’s disease (n = 59) with hyperglycemia inadequately controlled by metformin or other oral antidiabetic agents. In these patients, there was a trend towards better control of HbA1c with incretin-based therapy versus insulin, particularly in patients with acromegaly (51). Since DPP4i at least indirectly influence the pathophysiology of pasireotide-induced hyperglycemia, they may also be considered as first-line treatment in some cases.

There are currently limited data on the use of SGLT-2 (sodium-glucose transport protein 2) inhibitors for the management of hyperglycemia in patients with acromegaly. In contrast to DPP-4i and GLP-1 RA, SGLT-2 inhibitors do not affect the pasireotide-induced decreased incretin effect and therefore have presumably less impact on the specific pathophysiology. Moreover, it is important to note that acromegaly patients are at an increased risk of developing diabetic ketoacidosis (DKA), a common complication of SGLT-2 inhibitors (52–54). Therefore, SGLT-2 inhibitors are not typically recommended for patients with acromegaly. The shift in insulin/glucagon ratio as observed in pasireotide treatment is thought to be especially prone to this side-effect, warranting greater caution (55). However, patients safely treated with pasireotide and SGLT-2 inhibitors have been reported (56). In current T2DM guidelines, SGLT-2 inhibitors may be considered for certain patient groups, especially those with heart failure and renal disease. In these patients, SGLT-2 inhibitors with primary evidence of reducing chronic kidney disease (CKD) progression and heart failure may be an option; accordingly, acromegaly patients harboring these characteristics could benefit from this treatment, but it should be carefully weighed against the potential risk of DKA (49, 55).

The addition of insulin may be considered, but it should ideally be used as an adjunct to metformin and at least one other therapeutic agent. The current consensus report by the American Diabetes Association (ADA) and the European Association for the Study of Diabetes (EASD) advises the consideration of a GLP-1 RA prior to initiation of insulin therapy (49). It’s important to note, in the context of insulin therapy, that several challenges may arise. One of the significant issues is the risk of hypoglycemia, particularly if injection intervals are not consistently maintained or if insulin doses are not properly adjusted to match carbohydrate intake. Additionally, a notable disadvantage of insulin therapy is the potential for weight gain. This risk factor should be carefully weighed, especially in patients for whom weight management is a critical component of their overall health strategy.

**Figure 2:**
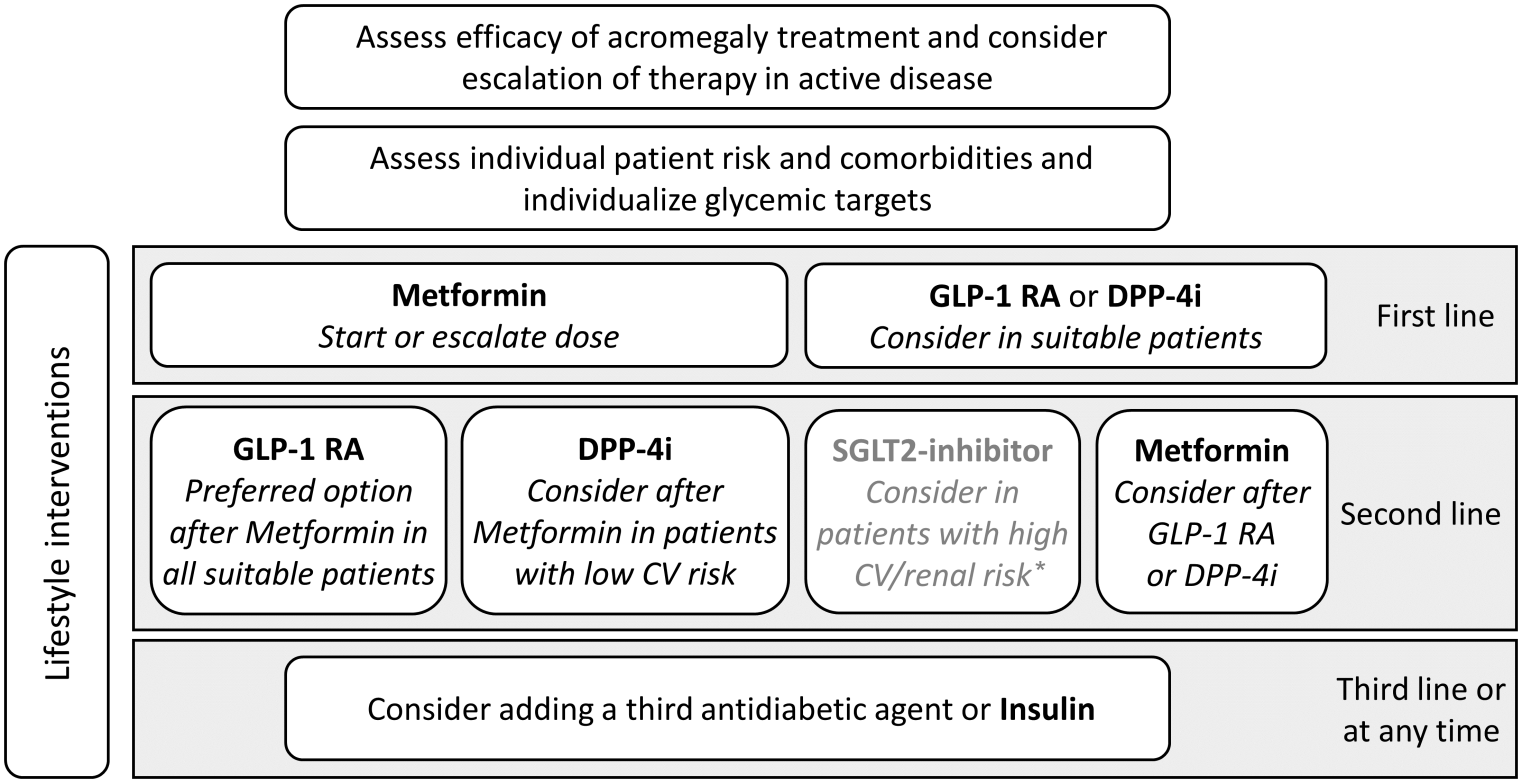
Recommendations on the treatment of hyperglycemia and diabetes in patients with acromegaly treated with pasireotide. *carefully weigh potential benefits on heart failure and renal disease against potential risk of diabetic ketoacidosis (CV, Cardiovascular; DPP-4i, Dipeptidyl peptidase 4 inhibitor; GLP-1 RA, Glucagon-like peptide-1 receptor agonist; SGLT-2, sodium-glucose transport protein 2; T2DM, Type 2 diabetes mellitus).

A summary of the recommendations on the treatment of hyperglycemia and diabetes in patients with acromegaly treated with pasireotide are displayed in **Figure 2**.

While co-agonists of GLP-1 and GIP (glucose-dependent insulinotropic polypeptide), such as tirzepatide, present a potentially intriguing therapeutic option for the future, the current lack of extensive precludes a definitive recommendation for their use in managing pasireotide-induced hyperglycemia. However, it is practical to consider these dual incretin agonists as a more feasible alternative to insulin therapy in this context. While acknowledging the limited data, tirzepatide should be included in the discussion about GLP-1 RA as a promising option for patients experiencing hyperglycemia due to pasireotide

In the treatment of hyperglycemia when pasireotide therapy is paused or discontinued, it is useful to distinguish between treatment-associated or pre-existing disease. In the case of pre-existing disease antidiabetic therapy may need to be continued as before the start of pasireotide therapy. Any pasireotide-related hyperglycemia can be expected to improve after pasireotide therapy is stopped. In this case, it may be appropriate to reduce or discontinue antihyperglycemic treatment. Metformin and other antidiabetic medications with low hypoglycemic risk can be discontinued when individual HbA1c targets are reached. Patients receiving insulin therapy are at higher risk of developing hypoglycemia, but usually perform regular SMBG measurements so that these patients can reduce insulin when their target levels are reached. If HbA1c levels are in or near the normal range, de-escalation of antidiabetic treatment should be considered.

There seems to be no clear relationship between the dose of pasireotide and the risk of hyperglycemia, and even low doses can cause worsening of glycemic control (22). Thus, treatment decisions should be made irrespective of the pasireotide dose and primarily guided by blood glucose assessment and individualized targets.

It should also be considered that acromegaly patients with hyperglycemia are often younger than T2DM patients. In clinical studies and real-world cohorts, the mean age of acromegaly patients is often between 40 and 50 years, with non-negligible proportions of female patients of reproductive age (13, 14, 32, 47). This particular risk structure of acromegaly patients should be considered regarding the choice of therapy. In case of pregnancy, management of diabetes should be in concordance with the current ADA guidelines (57).

In summary, we emphasize the importance of adequate control of acromegaly, excellent diabetes education, nutrition and lifestyle counseling as the foundation of management of acromegaly and hyperglycemic adverse events under pasireotide. Importantly, our expert recommendations address the specific pathophysiology of pasireotide-induced hyperglycemia by recommending the incretin-based therapeutics DPP-4i and GLP-1 RA as first-line therapy as an alternative to metformin in all appropriate patients when pharmacotherapy is needed.

## 7 Summary

The second-generation SSA pasireotide has been attributed with potentially greater clinical efficacy in acromegaly compared to first-generation SSA (9, 11, 12). However, several clinical trials emphasize the occurrence of hyperglycemia-related adverse events and diabetes in acromegaly patients treated with pasireotide (13–16, 24, 26, 27, 29–33). Patients with acromegaly due to somatotroph adenoma with a high expression ratio of the somatostatin receptors SSTR5/SSTR2, a hyperintense T2-weighted MRI signal, sparse granulation, low AIP expression, high AIP mutation status, low expression of E-cadherin and high expression of Ki-67 are considered good candidates to benefit from pasireotide treatment (12, 18, 39–45). Despite the potential of these indicators, limited comprehensive studies and routine clinical practices make it challenging for clinicians to use these markers for patient selection, leading to a reliance on imaging and patient history.

Acromegaly patients who are metabolically healthy, young (<40), and have a BMI <30 kg/m² are considered to be at low risk for pasireotide-associated hyperglycemia (47). Blood glucose tests should be performed prior to initiation of pasireotide to stratify patients by baseline risk for worsening of glucose control.

We recommend close glucose monitoring of fasting and post-prandial glucose levels during the first three month after initiating pasireotide treatment. Baseline risk determines our recommendation for the frequency of self-monitoring of blood glucose (SMBG) as outlined in **Figure 1**.

Adequate treatment of acromegaly is the priority in all patients. As a preventive measure or after the occurrence of hyperglycemic events, diabetes education, nutrition and lifestyle guidance should be provided to all patients regardless of whether they are achieving their individual blood glucose targets.

When pharmacotherapy for hyperglycemia is necessary, our expert recommendations address the specific pathophysiology of pasireotide-induced hyperglycemia by recommending the incretin-based therapeutics GLP-1 RA and DPP-4i as first-line therapy in all appropriate patients as an alternative to metformin. **Figure 2** summarizes key recommendations on our suggested treatment approach.

## Data availability statement

The original contributions presented in the study are included in the article/supplementary material. Further inquiries can be directed to the corresponding author.

## Author contributions

SS: Conceptualization, Formal Analysis, Funding acquisition, Investigation, Methodology, Project administration, Supervision, Writing – original draft, Writing – review & editing, Data curation. SM: Data curation, Investigation, Methodology, Supervision, Writing – original draft, Writing – review & editing. JG: Data curation, Investigation, Writing – original draft, Writing – review & editing. JF: Data curation, Investigation, Writing – original draft, Writing – review & editing. KS: Data curation, Investigation, Writing – original draft, Writing – review & editing. JS: Data curation, Investigation, Methodology, Supervision, Writing – original draft, Writing – review & editing. BV: Conceptualization, Data curation, Investigation, Methodology, Supervision, Writing – original draft, Writing – review & editing.
